# Size control in growing yeast and mammalian cells

**DOI:** 10.1186/1742-4682-1-12

**Published:** 2004-11-16

**Authors:** Akos Sveiczer, Bela Novak, J Murdoch Mitchison

**Affiliations:** 1 Department of Agricultural Chemical Technology, Budapest University of Technology and Economics, 1111 Budapest, Szt. Gellert ter 4, Hungary; 2 Molecular Network Dynamics Research Group of Hungarian Academy of Sciences, Budapest University of Technology and Economics, 1111 Budapest, Szt. Gellert ter 4, Hungary; 3 Institute of Cell, Animal and Population Biology, University of Edinburgh, Ashworth Laboratories, West Mains Rd, Edinburgh EH9 3JT, Scotland, UK

**Keywords:** cell size, size control, checkpoint, fission yeast, cultured Schwann cells

## Abstract

**Background:**

In a recent publication it was claimed that cultured mammalian cells, in contrast to yeasts, maintain a constant size distribution in the population without a size checkpoint. This inference may be challengeable.

**Results:**

(1) It is argued that "weak" size control implies the existence of a checkpoint, and unfortunately the technique used by Conlon and Raff might obscure such a weak mechanism. (2) Previous investigations of size control in yeasts have shown that individual cell data, rather than means and variances of cell populations, are prerequisites for reliable interpretation. (3) No experimental data so far obtained suggest that in any cell culture a linear growth pattern in cell mass can maintain size homeostasis on its own without size control. (4) Studies on fission yeast mutants indicate that the molecular mechanisms of size control vary with genetic background, implying that no single mechanism is likely to apply to any cell type, including cultured mammalian cells, under all conditions.

**Conclusion:**

The claim that cultured mammalian cells maintain size homeostasis without a checkpoint needs to be re-evaluated by measurements on individual cells.

## Introduction

Conlon and Raff [1] recently stated that constant size distribution is maintained in a culture of proliferating rat Schwann cells without the need for any size checkpoint, in contrast to the situation in yeast. Before discussing their conclusion, it is important to consider their techniques and nomenclature. They used an electronic (Coulter) counter to measure the mean and variation of cell volume at intervals in whole cell populations. In fission yeast, the most detailed study to date involved the measurement of lengths (proportional to volume) of *single *growing cells [2]. This is a vital matter that is not fully appreciated.

There is a long history of size measurements on fission yeast and the involvement of size in the control of the cell cycle, see e.g. [3-5], but only recently has the term "size control" come into use [2,6,7]. Although convenient, the phrase is something of a cover-all, since the nature of "size control" varies in fission yeast and the term is unlikely to denote exactly the same phenomena in mammalian cells. Moreover, mammalian cells normally form tissues, where greater emphasis has to be placed on external factors. However, size control operates in all systems where it has been possible to examine individual cells through their cell cycle. Its nature is likely to be different in each, although this will only become clear when its molecular basis is understood [6].

## Discussion

### The "evidence against size checkpoints" in cultured Schwann cells: an assessment

One of the two cases cited by Conlon and Raff as evidence for the lack of a size checkpoint is the slow change in size when cells are stimulated by fresh medium [1]. The reason we do not regard this as strong evidence comes from data on fission yeast. When either cycle time or total length extension is plotted as a function of birth length, the negative slope of the regression line is large in wild-type cells, implying that size control is "strong" and deviations from the average will be corrected within a single cycle [2]. But the slope can be less, as for example in diploids, and then the control becomes "weak", since deviations will not be corrected within one cycle. Without the support of single cell data, it is nevertheless possible to imagine the presence of a weak size control in mammalian cells, homologous to that in yeast, exerting a slow but important action over several cycles. But can such a weak size control be considered a checkpoint? This entails another semantic or philosophical problem, since "checkpoint" is as ambiguous as "size control". Those who regard very sharp responses (e.g. all or none) as a criterion would argue that a weak mechanism is not a checkpoint. However, since it fulfils its function by ensuring homeostasis in a cell population, we believe that a weak size control should also be considered a checkpoint.

Another problem in [1] is the increasing size of quiescent cells blocked in S phase by aphidicolin and stimulated into growth. The mean cell volume increased linearly five-fold over 100 h. A similar if less marked increase was also found in total protein. In addition, the valuable observation was made that the rates of protein synthesis and degradation increased with size. It is almost certainly true that size-dependent synthesis and degradation would not result in a size-independent pattern of protein accumulation. In the simplest case, if the rates of both protein synthesis and degradation are linear functions of total protein (mass), protein content will show a size-dependent exponential dependence on time. Therefore, further measurements and analyses need to be carried out to resolve this discrepancy. Moreover, it follows from the hypothesis of Brooks [8] that, in the absence of size control, an exponential growth of cell volume results in a continuous increase in the dispersion of cell size at division; in contrast, linear growth would not increase the dispersion in consecutive cycles, and individual cell sizes would converge towards the mean after perturbations. To date, however, no experimental evidence has been published to show that a cell culture can maintain homeostasis by linear growth *without *a size checkpoint. It is plausible that this might happen because of the general need for co-ordination between the cytoplasmic and chromosome cycles, but we are not convinced that linear growth on its own would meet this requirement. In the case of fission yeast, there is evidence that linear growth without a size checkpoint cannot maintain homeostasis in the culture (see below).

### Results of similar experiments with fission yeast

Related observations from fission yeast show that normal size control is abolished in the type of block experiment used in [1]; however this might be apparent only after release. This was first shown by Fantes [3] and was later expanded by us [2]. The time-scale, however, was shorter than in mammalian cells. It is also true, as Conlon and Raff [1] point out, that the pattern of total protein and RNA content in several yeast *cdc *mutants was approximately exponential over a longer period after this type of block. This result comes from an early paper and depends on measurements of absolute amounts of protein and RNA/ml [5]. There is not much evidence from the far more accurate method of measuring synthesis rates by pulses of radioactive precursor, but such evidence as exists fails to support the conclusion. In the widely used mutant *cdc2-33*, the rate of protein synthesis reaches a plateau after 4 h [9]. Also, only fairly minor changes in protein and RNA synthesis rates occur over 7 h in the mutant *cdc13 *[10]. Because linear growth was measured in mammalian cells during a long S phase block [1], we have another objection to make here. The fission yeast cell cycle can be considered as linear segments of volume growth with points (called rate change-points) where there are changes in growth rate [11], which may be partly associated with a gene-dosage effect [2]. If a similar mechanism operates in mammalian cells during the normal cycle, it could never be observed when replication is arrested.

### Genetic background and size control in fission yeast

There are other points about the fission yeast experiments that are relevant to size control, which is almost certainly affected by genetic background. The main rate change point (an effect of gene-dosage, see above) is shifted in position in *wee *mutants (S phase is also shifted), but this size control is a strong one, albeit weaker in diploids [2]. There appears to be a different mechanism in the strange double mutant *wee1-50 cdc25Δ *[12]. It seems improbable that all these variations involve the same molecular mechanisms; rather, they suggest a variety of options in the nature and positions of size controls. But since we do not understand the molecular mechanisms involved, these matters remain to be resolved.

Another point is that the normal rules of the cycle can be broken temporarily, but not for long. For example, mammalian cells can certainly grow without entering S phase [6]; and the very large cells of early embryos only develop a size control after a number of cycles [13]. Block and release protocols can be used to induce synchrony in *cdc *mutants of fission yeast, and size control is lost for some cycles (but not forever), as indicated by the lack of negative correlation between length extension and birth length [2,3]. However, if size control has been permanently lost, as in the case of the double mutant *wee1-50 rum1Δ*, the cells lose viability after a few generations [14] despite the near-linearity of their length growth pattern [2]. According to the hypothesis of Brooks [8], cell size should be convergent in this case after some generations, leading to size homeostasis. However, the mathematical solution seems not to be applicable for the yeast cells, which rather die.

## Conclusions

To return to mammalian cells: our view is that the best way forward is to try to devise ways of following individual cells through the cycle. It is easy for us to say this, since we have had the great advantage of working with regular shaped yeast cells that stay still and grow steadily on agar. The mammalian cells situation is far more difficult, but there is reasonable hope that it can be solved by methods of tracking individual cells such as fibroblasts by a semi-automatic method and measuring their dry mass by interferometry [15]. The main problem with the technique of Conlon and Raff is that it cannot distinguish between a weak (but existing!) size control and its total lack. Very recently, similar experiments have been done with erythroblasts and fibroblasts from different vertebrates, and the results seem to suggest the existence of a strong size control in every case [16]. Our present knowledge that the existence of size control (either weak or strong) seems to be general, suggests that rat Schwann cells are probably not exceptions above the rule, but they rather have a weak size control in spite of the conclusions of Conlon and Raff [1]. To choose the correct interpretation between the two possible ones is a challenge for the future.
